# Supplementary material for: Yiai Fuzheng decoction inhibits triple-negative breast cancer by remodeling the immune microenvironment
Source: Front Immunol. 2025 Sep 30;16:1615631. doi: 10.3389/fimmu.2025.1615631 (PMC12518410; doi:10.3389/fimmu.2025.1615631)
Supplement: Supplementary file 2 [file DataSheet1.zip › Supplementary File 2/Treatment/C_vs_D_vs_H/02_PCA/All_Groups/PCA_Score_with_Boxplot_with_Points.pdf]

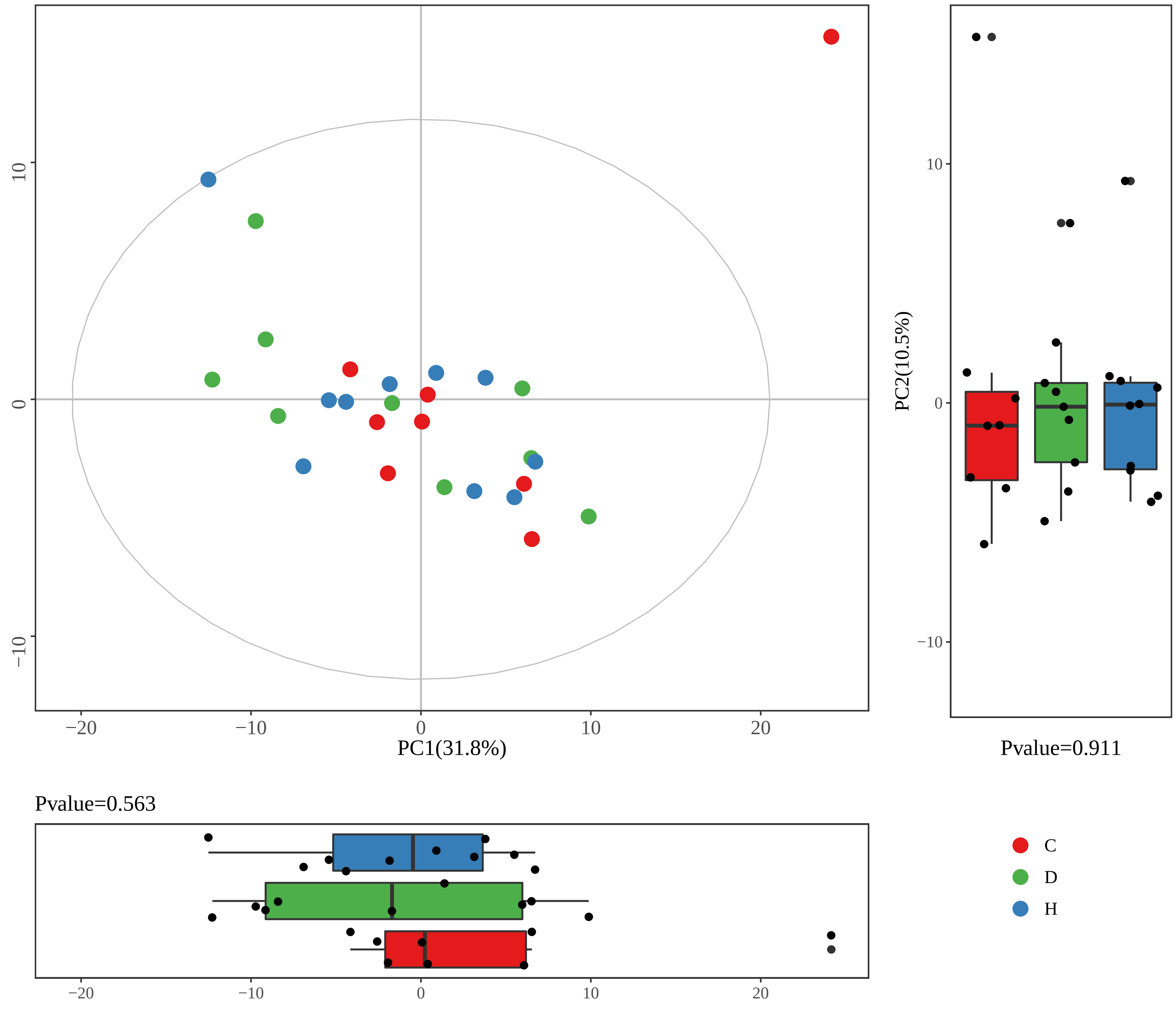

PC1(31.8%)
PC2(10.5%)
Pvalue=0.911
Pvalue=0.563
−20
−10
0
10
20
C
D
H
